# Incidence, Associated Factors, and Outcome of Delirium among Patients Admitted to ICUs in Oman

**DOI:** 10.1155/2022/4692483

**Published:** 2022-10-04

**Authors:** Rasha Khamis Al-Hoodar, Eilean Rathinasamy Lazarus, Omar Al Omari, Omar Al Zaabi

**Affiliations:** College of Nursing, Sultan Qaboos University, Muscat, Oman

## Abstract

**Background:**

The incidence of delirium is high up to 46.3% among patients admitted to ICU. Delirium is linked to negative patient outcomes like increased duration of mechanical ventilation use, prolonged ICU stay, increased mortality rate, and healthcare costs. Despite the importance of delirium and its consequences that are significant, there is a scarcity of studies which explored delirium in Oman.

**Objectives:**

This study was conducted to assess the incidence of delirium, the association between the selected predisposing factors and precipitating factors with delirium, determine the predicators of delirium, and evaluate its impacts on ICU mortality and ICU length of stay among ICU patients in Oman.

**Methods:**

A multicenter prospective observational design was used. A total of 153 patients were assessed two-times a day by bedside ICU nurses through the Intensive Care Delirium Screening Checklist (ICDSC).

**Results:**

The results revealed that the delirium incidence was 26.1%. Regression analysis showed that sepsis, metabolic acidosis, nasogastric tube use, and APACHE II score were independent predictors for delirium among ICU patients in Oman and delirium had significant impacts on ICU length of stay and mortality rate.

**Conclusion:**

Delirium is common among ICU patients and it is associated with negative consequences. Multidisciplinary prevention strategies should be implemented to identify and treat the modifiable risk factors.

## 1. Introduction

Delirium has a major economic impact on the health care system. In the United State of America, the total health care expenditures associated with delirium are ranged from $38 to $152 billion each year, including costs associated with readmission, falls, and long-term care [1, 2] and the care costs of one patient with delirium increased their total care cost by about $600 per day and $18,000 over 30 days due to an increase in service use, including bed-related expenses, laboratory and diagnostic radiology costs, and pharmacy costs [3]. High incidence of delirium up to 46.3% among patients admitted to ICU had been reported [4], and strategies to avoid and prevent delirium has been a central topic of research. The society of Critical Care Medicine published clinical practice guidelines for the management of delirium in the intensive care unit and described validated delirium screening tools for daily use for critically ill patients [5]. This guideline was updated in 2018, strongly recommended on assessing all adult ICU patients for delirium every eight hours using a valid and reliable delirium assessment tool [6]. Daily delirium assessment is effective strategy to prevent delirium in 30% to 40% of cases through early detection of delirium symptoms [7]. Delirium has been defined as an acute neuropsychiatric condition characterized by alteration in consciousness, attention, and inability to focus that develops over a short period of time, associated with an impairment of recent and immediate memory with fluctuating course, due to an underlying medical condition, substance intoxication or withdrawal, or due to different etiologies [8]. Moreover, the International Classification of Diseases, 11^th^ Revision defined delirium as acute onset of reduced arousal, cognitive deficits, disturbances of attention, motor activity, and sleep-wake [9].

Over the past decade, several tools have been developed to assess delirium among hospitalized patients including Confusion Assessment Method for the Intensive Care Unit (CAM-ICU), Cognitive Test for Delirium, Delirium Detection Score, Intensive Care Delirium Screening Checklist (ICDSC), and Nursing Delirium Screening Scale [10]. Based on the analysis of psychometric properties of the tools, CAM-ICU and the ICDSC are the most valid and reliable delirium assessment tools for critically ill patients [10].

The exact pathophysiology of delirium is not fully understood, but the major cause for delirium includes ischemic brain injury, an imbalance of neurotransmitters and peripheral inflammation of the brain [11]. Previous research revealed that the etiology of delirium is multifactorial and identified several factors that negatively contribute to delirium development, divided into the preexisting condition of the patient, acute condition of the patient, and iatrogenic or environmental factors [7, 12]. The baseline factors included age [4], severity of illness [13],and presence of comorbidities [14]. While the hospital factors were sedation use [14], vasopressors use [15], length of ICU stay [4], mechanical ventilation use, presence of hypoxia, fever, raised levels of bilirubin and creatinine [16], physical restraints use, and sepsis [17].

Previous studies reported that the incidence of delirium range between17.3% [18] and 54.9% [19]. Previous studies have investigated the effects of delirium. It has been reported that delirium has significant impacts on patient safety and is a strong independent predictor for an increased risk of falling [20]. Patients with delirium are more vulnerable to removing invasive catheters, endotracheal tubes, and urinary catheters than their non-delirious patients [14]. Delirium is associated with prolonged mechanical ventilation use, extended hospitalization, and increased morbidity, and mortality rates [2]. Moreover, it not causes acute adverse consequences but also caused long-term negative impacts on the quality of patient life [21] and long-term cognitive impairment among ICU survivors [22].

At the international level, there are many tools used to screen ICU patients for delirium. There is no specific protocol that guides health care professionals in Oman to assess patients for delirium at a national level. Despite the importance of delirium and its consequences that are significant, there is a scarcity of studies which explored delirium in Oman and little is known about the characteristics of patients having delirium, delirium incidence and the relationship between delirium and patient outcomes. Therefore, aiming to fill this gap, this study is the first of its kind in Oman that investigated delirium incidence, risk factors, and outcomes associated with delirium. It provided important insights about delirium in the critical care units and the intention to implement a screening tool.

Oman and the other Gulf countries share a unique culture and ethnic characteristics compared to other developed and developing countries. However, no much studies available representing their culture and its influence on the occurrence of delirium. Identifying the level of in this study will help healthcare professionals in Oman develop a prediction model to help them predict which patients are at higher risk of developing delirium.

## 2. Materials and Methods

### 2.1. Study Design and Purpose

A prospective observational design was used to (1) assess the incidence of delirium, (2) identify the association between the selected variables including predisposing factors, precipitating factors with delirium, (3) determine to what extent the selected variables predict delirium, and (4) determine the impacts of delirium on ICU length of stay and ICU mortality among ICU patient in selected hospitals in Oman.

### 2.2. Sample and Setting

The study was conducted in two major governmental hospitals in Muscat, the capital city of Oman. There are seven governates in Oman. These two big government hospitals in the capital city receive patients from all these governorates due to nonavailability of high sophisticated and complex ICUs in other areas. A sample of 153 patients who admitted to Intensive Care Units including adult ICU, Coronary Care Unit, and post cardiac surgery Unit, over three months from September to December 2020 and met the inclusion criteria were recruited. Sample size was calculated based on the number of independent variable (*m*) which used in the logistic regression model with 5% of significance and 80% power. The estimated sample size was 150 participants. The nonprobability convenience sampling method was used to recruit participants who are fit with the inclusion criteria during the study timeline.

### 2.3. Measurements

#### 2.3.1. Intensive Care Delirium Screening Checklist (ICDSC)

In the current study, the Intensive Care Delirium Screening Checklist was used to assess patients for delirium. It was developed by Bergeron and colleagues [23]. It consisted of eight items based on DSM criteria, which are altered level of consciousness, inattention, disorientation, hallucination/delusions/psychosis, psychomotor agitation or retardation, inappropriate speech or mood, sleep wake/cycle disturbance, and symptom fluctuation [23]. It can be applied by ICU nurses or medical staff without formal psychiatric training and it is suitable to screen patients in busy ICUs [23]. ICDSC relies on the observational method in order to identify these symptoms and do not require to ask patient to answer any questions [23]. Patients were given one point for each symptom that manifests within the time period and zero points if the symptom did not manifest [23]. For any items that cannot be assessed, scored no point. A score of 4 or more out of a possible score of 8 was found to be indicative of delirium. If the patient scores >4, notify the physician [23]. The psychometric properties for ICDSC showed interrater reliability was high and found to be 0.947 [24]. It showed a good diagnostic accuracy with a sensitivity of 81.0%, a specificity of 87.7%, and it had good internal consistency, with Cronbach *α* of 0.839 [25]. In this study, English version was used. Permission to use the tool was taken.

#### 2.3.2. Acute Physiology and Chronic Health Evaluation II (APACHEII)

APACHEII score, a measure of severity of disease, was introduced in 1985 by Knaus et al. [26]. It is point score calculated based on initial values of 12 routine physiological points, age points, and chronic health points, measured during the first 24 h of ICU admission [26]. Each parameter was assigned from 0 to 4, with 0 being normal and four being the most abnormal. With the minimum score of zero and maximum score of 71, increasing score is associated with increasing risk of hospital death [26]. It has a sensitivity of 87.5% and a specificity of 79.0% as predictor for ICU mortality [27].

#### 2.3.3. Sequential Organ Failure Assessment (SOFA) Score

The SOFA score was developed following a consensus meeting in 1994 by Working Group on Sepsis-Related Problems of the European Society of Intensive Care Medicine [28]. It describes the degree of organ dysfunction in patients quantitively and describes a sequence of complications in the critically ill [28]. The score based on six items, one for each organ systems, respiratory, cardiovascular, hepatic, coagulation, renal, and neurological systems. Each scored from 0 to 4 and total score ranged from 0 to 24 points, increasing score indicating worsening of organ dysfunction [28]. It had 85% sensitivity and 73.9% specificity for predicting hospital mortality [29].

#### 2.3.4. Simplified Acute Physiology Score (SAPS) II

The SAPSII score was introduced in 1984 as an alternative to APACHE scoring, and published by Le Gall et al. [30]. It used to estimate hospital mortality [30]. It included 17 variables–12 physiological variables, age, type of admission (scheduled surgical, unscheduled surgical, or medical), and 3 variables related to underlying disease: acquired immunodeficiency syndrome, metastatic cancer, and hematological malignancy [30]. The score is sum of points which assigned for each variable vary from 0 to 3 (for temperature) and from 0 to 26 for the Glasgow coma scale [30]. The worst value during the first 24 hours in the ICU admission is taken into account [30]. It has good discrimination, calibration, and power to predict deaths on ICU [31]. It has a sensitivity of 87.3% and a specificity of 89.6% for hospital short-term mortality [32].

#### 2.3.5. Risk Factor Checklist

A risk factor checklist was specifically designed for this study based on the review of literature with respect to risk factors for delirium among ICU patients. The variables which have been incorporated in the checklist include age, gender, smoking, presence of comorbidity, Pre ICU emergency surgery or trauma, APACHEII score, SOFA score, sedation use, sepsis, mechanical ventilator use, serum bilirubin, serum creatinine, metabolic acidosis, bladder catheter use, and nasogastric tube use.

### 2.4. Data Collection Procedure

The investigators approached the in-charges of the ICUs and provided them with overview of the study purposes, methods, and significance. Then, training in delirium assessment using ICDSC started for two weeks. Following this, the researchers identified participants who met the eligibility criteria (admitted a least 24 H, aged 18 years or above, understand or speak either Arabic or English, transferred from another hospital or from another ICU or ward). Then, the researchers or the nurse was introduced himself/herself to the patients and relatives and explained the study purpose and procedures to obtain their permission to participate in this study. A package comprising an information sheet, consent sheet was given to each eligible participant. After 24 H of admission, delirium was assessed by nurse or researcher using ICDSC for patients who agreed to participate in the study and retrieved the patient's data from the records. Nurses collected the data collection sheet to help them. Patients with an ICDSC score of 4 or greater were classified as delirium-positive [23]. Positive cases were reported to the ICU physician and necessary consultation services was provided when requested.

For each enrolled patient, about 15 variables were collected from electronic patient record (EPR) including demographics, data concerning the past medical history (Presence of comorbidities, smoking), and type of admission. Risk factor checklist was checked for sedation use, mechanical ventilator use, sepsis, and bladder catheter use and nasogastric tube insertion within the last 24 h. In addition, APACHE II, SOFA score, and SAPS II score were calculated using the worst laboratory values within 24 h of ICU admission. The mortality rate as calculated according to the Simplified Acute Physiology Score II (SAPS II). Moreover, the length of ICU stay was calculated in days from the day of admission to the day of discharge for ICU.

### 2.5. Ethical Consideration

Permissions to undertake the study were granted from the ethical committees of the collage of nursing, Ministry of Health (MOH) and Sultan Qaboos University Hospital. In addition, permission to use the tool was obtained. Written consent was obtained from all participants. Participation was voluntary and no identification data were collected.

### 2.6. Statistical Analysis

Statistical Package for Social Science (SPSS) Software program version 23 was used to manage and analyze the data. Data cleaning and verification was done prior to conduct the analysis. Continuous variables were displayed as mean and standard deviation (SD). Nominal variables were shown as a number and percentage and were analyzed using contingency tables and *χ* test. Bivariate associations between continuous variables were investigated with point-biserial correlation. Binary logistic regression analysis was used to examine the independent association between the explored variables and the presence of delirium. All the independent variables that were correlated with delirium in the bivariate level were entered into the initial regression model. For all tests, statistical significance was set at an alpha level of *p* = 0.05.

## 3. Results

The study started from mid- September to mid-December 2020. During the study period, 210 patients were admitted to ICUs. A total of 57 patients were excluded because 27 patients with a RASS score <−3 during the entire study period and 10 patients stayed less than 24 H in the unit. An additional, five patients were excluded because they are neuropsychiatric and 15 patients were excluded because they underwent neurosurgery. Finally, a total of 153 patients participated in the study from both hospital, SQUH and RH. Out of 153 participants, 40 developed delirium (26.1%). Of these, 98 were male (64.1%) and 55 were female (35.9%). The mean age of the participants was 53 years (SD 19.6). About 88 were reported with medical conditions (57.5%) and majority of the participants had co-morbidity165 (92.2%). Most of them were nonsmokers 128 (83.7%).

During the study periods, majority of the participants required a mechanical ventilator 109(71.2%). Approximately, half of them were on one vasopressor 79(51.6%) and 45 of them were on one sedative medication (29.4%). The mean APACHEII score of the participants was 18 (*SD* = 5.6) which is the severity of the illness, while the mean score of organ dysfunction (SOFA) was 8 (*SD* = 3.2). The other characteristics of participants are summarized in Table 1.

### 3.1. Correlation between Selected Predisposing Factors and Precipitating Factors with Delirium

A point-biserial correlation was computed to determine the relationship between the selected sociodemographic and clinical variables including age, comorbidity, APACHEII score, SOFA score, sedation use, bilirubin level, creatinine level, and delirium. The results revealed that positive correlation between APACHEII score, SOFA score, sedation use, high creatinine level, and delirium.  Table 2 describes the correlation results.

Chi-square analysis was performed to assess the relationship between selected categorical variables including gender, smoking, emergency surgery or trauma, sepsis, ventilator use, metabolic acidosis, bladder catheter use, nasogastric tube use, and delirium. The results identified significant relationships between emergency surgery or trauma, sepsis, ventilator use, metabolic acidosis, bladder catheter use, nasogastric tube use, and delirium.  Table 3 describes the correlation results.

### 3.2. Predicators of Delirium

Binary logistic regression analysis was conducted to identify the significant predicators for developing ICU delirium. The enter method was used to test for significant predictors. All the independent variables that were correlated with delirium in the bivariate level were entered into the initial regression model. Then, only the variables that were correlated in the initial regression model were entered into the regression model. The overall success perdition rate of the model was 86.3%. The results indicated that there were positive relationships between sepsis, metabolic acidosis, nasogastric tube use, APACHE II score, and delirium and those were predicted delirium among ICU patients in selected hospitals in Oman (*R*^2=^0.519, adjusted *R*^2=^0.519, *p* *≤* 0.01).  Table 4 details the regression results.

### 3.3. Impact of Delirium on ICU Mortality and ICU Length of Stay

A point-biserial correlation was computed to assess the relationship between ICU mortality and ICU length of stay and delirium. The results indicated a positive correlation between predicated ICU mortality rate, ICU length of stay, and delirium.

## 4. Discussion

To the best of the knowledge of the researcher, this is the first study to identify the incidence of delirium, explore associated factors of delirium, and evaluate its impact on ICU length of stay and ICU mortality among ICU patients in Oman. The results showed that the incidence of delirium among ICU patients is 26.1%, which demonstrates a lower incidence than reported results in previous studies that conducted in Tunisia [14], Saudi Arabia [18], and Italy [33]. The lower incidence estimated in the current study may be related to application of some delirium prevention interventions through pharmacological and nonpharmacological interventions like a sedation weaning, pain management and early mobilization. Theses interventions are very effective in reducing incidence of delirium through targeting the risk factors of delirium.

This variation in the incidence of delirium in the literature related to variations in patient populations, sample size, study setting, source of sample, and data collection method. For example, the Canadian study was conducted in multisetting [17] while other studies were conducted in a single setting in China (*n* *=* 320) ([34] Czech Republic (*n* *=* 332) [15] and Szczecin (*n* *=* 1,797) [19] with large sample size. There is a possibility that the character of the study sample had a significant impact on delirium incidence.

The results found that no difference in age between the participants with and without delirium. This result was not in line with other studies, which had shown significant association between increased age and delirium [14, 35, 36]. This discrepancy might be related to patient selection and type of ICU studies as this study included participants admitted to neurosurgery ICU [36]. Results from the current study showed that smoking had no association with delirium, corresponding to the findings of previous study [37]. The reason may relate to improper documentation of the smoking status in the hospital system.

APACHE II and SOFA scores represent illness severity and organ dysfunction, respectively. In this study, the participants who developed delirium had higher APACHE II and SOFA scores than those without delirium. Patients with severe illness and organ dysfunction may face a higher risk of developing delirium and indicating that delirium was associated with serious conditions. This study identifies sedative drugs use as precipitating factors as reported in prior studies [18, 38–41]. The possible cause is disruption of neurotransmitter system due to depression of the central nervous system [42]. The current study demonstrates a correlation between mechanical ventilator use and delirium (*p* ≤ 0.001). The possible explanation of the association between delirium and mechanical ventilator is use of sedation [18]. The second possible explanation is sleep deprivation [43] caused by pain, discomfort, anxiety, noise, light, and ICU care-related activities [44]. Previous studies supported that ventilator use is a significant risk factor for developing delirium [4, 36, 38, 45, 46].

Results of the current study are consistent with the previous studies [13, 14, 47, 48] where metabolic acidosis was significantly associated with delirium development. The underlying cause was reduction in acetylcholine activity in the brain due to electrolyte imbalance [49]. Results indicated that presence of renal impairment in term of high creatinine correlated positively with delirium. The results are consistent with the literature [50–52]. This result builds on the existing evidence on effects of accumulation of waste products in case of kidney impairment on the brain through inducing inflammation and release of pro-inflammatory markers that may precipitate delirium development [53].

Sepsis was found to have significant association with the development of delirium. The finding of the current study was in line with the hypothesis that sepsis cause activation of the systematic inflammatory response and release of cytokines and/or bacterial toxins that may disrupt the blood-brain barrier, causing hypoxia, cerebral metabolic changes, and inadequate cerebral perfusion, resulting in delirium [45]. Similar results about the significant association between ICU delirium and sepsis were reported in previous studies [17, 54, 55]. It was observed that presence of bladder catheter was associated with delirium, also observed in other studies [34, 56, 57]. This finding might be because the presence of the Foley catheter was associated with increased patient vulnerability to delirium. After all, it may lead to urinary infections and mobility restriction [58], precipitating occurrence of delirium.

Insertion of nasogastric tube is indicated for severity of the disease which is significant risk factor for delirium development. It is recommended, regular assessment for the need of lines to promote early removal. The current study showed that patients with nasogastric tube are more prone to develop delirium, similar to the results described in references [34, 48]. Insertion of nasogastric tube is indicated for the severity of the disease, which is a significant risk factor for delirium development. It is recommended, regular assessment for the need of lines to promote early removal.

In interpreting the results of this study, some limitation should be acknowledged. This study used a convenience sample and had a small sample size in the delirium group that may affect the statistical analysis which limits the generalizability of the results. Further research is needed to explore the impact of taking the preventive measures to reduce the incidence of delirium among ICU patients in Oman.

### 4.1. Highlights of the study

1. The results revealed that the delirium incidence was 26.1% in Oman
2. Sepsis, metabolic acidosis, nasogastric tube use, and APACHE II score were independent predictors for delirium among ICU patients in Oman
3. Delirium had significant impacts on ICU length of stay and mortality rate

## 5. Conclusion

Delirium is common disorder among ICU patients. It is a global concern because of its impacts on patient's outcomes and health care systems. It is a necessary to identify the significant risk factors that associated with delirium among ICU patients in Oman in order to plan for new strategies on delirium preventions through targeting the modifiable risk factors of delirium as prevention is better than cure.

## Figures and Tables

**Table 1:** Main clinical and demographic characteristics of the study participants(*N* = 153).

Variables	Total sample (*n* = 153)
*ICDSC a*	
Delirium	40 (26.1)
No delirium	113(73.9)
*Age (years) b*	53 ± 19.6 (18-78)

* Gender a*	
Male	98 (64.1)
Female	55 (35.9)

* Diagnosis a*	
Medical	88 (57.5)
Surgical	65 (42.5)

*Emergency surgery or trauma a*	
No	135 (88.2)
Yes	18 (11.8)

*Comorbidity a*	
None	12 (7.8)
One diseases	39 (25.5)
Two diseases	30 (19.6)
Three diseases	71 (46.4)
Six diseases	1 (0.7)

*Sepsis a*	
No	140 (91.5)
Yes	13 (8.5)

*Vasopressor use a*	
No drug	57 (37.3)
One drug	79 (51.6)
Three drug	11 (7.2)
Four drugs	6 (3.9)

*Ventilator use a*	
No	44 (28.8)
Yes	109 (71.2)

*Sedation a*	
No drug	50 (32.7)
One drug	45 (29.4)
Two drugs	58 (37.9)

*Metabolic acidosis a*	
No	88 (57.5)
Yes	65 (42.5)

*Bladder catheter use a*	
No	20 (13.2)
Yes	133 (86.9)

*Nasogastric tube use a*	
No	104 (68)
Yes	49 (32)
APACHEII (points) b	18 ± 5.6 (7–28)
SOFA (points) b	8 ± 3.2 (0–14)
SAPS II (points) b	48 ± 14.7 (22–85)
Bilirubin (mmol/L) b	20 ± 20 (3–86)
Creatinine (mmol/L) b	159 ± 158 (37–872)
Sodium (mEq/L) b	137 ± 8 (117–159)
ICU length of stay (days) b	6 ± 8 (2–51)

A, number (percentage). b, mean ± standard deviation (range).

**Table 2:** Summary of chi‐square test for delirium and the associated risk factors (*n* = 153).

Characteristics	Category	*Delirium*		*Chi-Square*	
		No (F)	Yes (F)	*χ* ^2^	*p* value
Gender	Male	77	21	3.14	0.087
	Female	36	19		
Smoking	No	93	35	0.58	0.454
	Yes	20	5		
Emergency surgery or trauma	No	101	34	0.55	0.460
	Yes	12	6		
Sepsis	No	108	32	9.22	0.002
	Yes	5	8		
Ventilator use	No	41	3	11.95	0.001
	Yes	72	37		
Metabolic acidosis	No	75	13	13.87	<0.01
	Yes	38	27		
Bladder catheter use	No	20	0	8.14	0.004
	Yes	93	40		
Nasogastric tube use	No	91	13	31.30	<0.01
	Yes	22	27		

*χ* ^2^ = Pearson chi-square value, *p* = significance level.

**Table 3:** Results of a point-biserial correlation test between delirium and continuous variables (*n* = 153)

Continuous variables	point-biserial correlation	*p* value
Age	−0.111	0.173
Comorbidity	0.040	0.623
APACHE II score	0.439	<0.01
SOFA score	0.354	<0.01
Sedation	0.193	0.017
Bilirubin	0.067	0.407
Creatinine	0.198	0.014

Continuous variable *N* pbis Mean (SD) *p* value. Age (years) 245 0.22 58.0 (10.5) < 0.05. Aortic cross clamp duration (minutes) 238 0.11 60.8 (27) > 0.05. Cardiopulmonary bypass pump duration (minutes) 238 0.07 96.7(33.9) > 0.05. Intraoperative crystalloids (mL) 245 0.02 2042.8 (241.9) > 0.05. Intraoperative blood/products (mL) 245 0.08 71.8 (305.7) > 0.05. Duration of surgery (minutes) 245 0.14 257.4 (63.6) < 0.05.

**Table 4:** Predictors of delirium in the study sample.

Variables	B	Wald	*p*	Exp (B)	95% Confidence limit
Sepsis	2.279	7.502	0.006	9.77	1.91–49.92
Metabolic acidosis	1.238	5.107	0.024	3.45	1.18–10.09
Nasogastric tube use	2.277	18.763	0.000	9.74	3.48–27.30
APACHEII score	0.200	11.486	0.001	1.22	1.09–1.37
Sedation use	0.087	0.065	0.799	1.09	0.56–2.12
Creatinine level	−0.003	1.726	0.189	1.00	0.99–1.00
Constant	−5.821	31.098	0.000	0.003	

Dependent Variable—ICU delirium.

## Data Availability

The data used to support the findings of this study are included within the article.

## References

[1] Leslie D. L., Leslie D. L., Marcantonio E. R., Zhang Y., Leo-Summers L., Inouye S. K. (2008). One-year health care costs associated with delirium in the elderly population. *Archives of Internal Medicine*.

[2] Salluh J. I. F., Wang H., Schneider E. B. (2015). Outcome of delirium in critically ill patients: systematic review and meta-analysis. *BMJ*.

[3] Vasilevskis E. E., Chandrasekhar R., Holtze C. H. (2018). The cost of ICU delirium and coma in the intensive care unit patient. *Medical Care*.

[4] Mori S., Takeda J. R. T., Carrara F. S. A., Cohrs C. R., Zanei S. S. V., Whitaker I. Y (2016). Incidence and factors related to delirium in an intensive care unit. *Revista da Escola de Enfermagem da USP*.

[5] Barr J., Fraser G. L., Puntillo K. (2013). Clinical practice guidelines for the management of pain, agitation, and delirium in adult patients in the intensive care unit. *Critical Care Medicine*.

[6] Devlin J. W., Skrobik Y., Gelinas C. (2018). Clinical practice guidelines for the prevention and management of pain, agitation/sedation, delirium, immobility, and sleep disruption in adult patients in the ICU. *Critical Care Medicine*.

[7] Inouye S. K., Westendorp R. G., Saczynski J. S. (2014). Delirium in elderly people. *The Lancet*.

[8] American Psychatric Association (2013). *Diagnostic and Statistical Manual of Mental Disorders (DSM-5®)*.

[9] Meagher D. J., Maclullich A. M., Laurila J. V. (2008). Defining delirium for the international classification of diseases, 11^th^ revision. *Journal of Psychosomatic Research*.

[10] Gelinas C., Berube M., Chevrier A. (2018). Delirium assessment tools for use in critically ill adults: a psychometric analysis and systematic review. *Critical Care Nurse*.

[11] Ali S., Patel M., Jabeen S. (2011). Insight into delirium. *Innovations in clinical neuroscience*.

[12] Jeste D. (2010). *Neurocognitive disorders. A Proposal from the DSM-5 Neurocognitive Disorders Work Group*.

[13] Gravante F., Giannarelli D., Pucci A. (2020). Prevalence and risk factors of delirium in the intensive care unit: an observational study. *Nursing in Critical Care*.

[14] Tilouche N., Hassen M. F., Ali H. B. S., Jaoued O., Gharbi R., El Atrous S. S (2018). Delirium in the intensive care unit: incidence, risk factors, and impact on outcome. *Indian Journal of Critical Care Medicine*.

[15] Kanova M., Sklienka P., Kula R., Burda M., Janoutova J. (2017). Incidence and risk factors for delirium development in ICU patients-a prospective observational study. *Biomedical Papers*.

[16] Jayaswal A. K., Sampath H., Soohinda G., Dutta S (2019). Delirium in medical intensive care units: incidence, subtypes, risk factors, and outcome. *Indian Journal of Psychiatry*.

[17] Duceppe M.-A., Williamson D. R., Elliott A. (2019). Modifiable risk factors for delirium in critically ill trauma patients: a multicenter prospective study. *Journal of Intensive Care Medicine*.

[18] Rasheed A. M., Amirah M., Abdallah M., Awajeh A. M., Parameaswari P. J., Al Harthy A (2019). Delirium incidence and risk factors in adult critically ill patients in Saudi Arabia. *Journal of Emergencies, Trauma, and Shock*.

[19] Kotfis K., Szylinska A., Listewnik M. (2018). Early delirium after cardiac surgery: an analysis of incidence and risk factors in elderly ((≥≥65 years) and very elderly ((≥≥80 years) patients. *Clinical Interventions in Aging*.

[20] Szewieczek J., Mazur K., Wilczyński K. (2016). Geriatric falls in the context of a hospital fall prevention program: delirium, low body mass index, and other risk factors. *Clinical Interventions in Aging*.

[21] Wang S., Mosher C., Perkins A. J. (2017). Post-intensive care unit psychiatric comorbidity and quality of life. *Journal of Hospital Medicine*.

[22] Wolters A. E., Peelen L. M., Veldhuijzen D. S. (2017). Long‐term self‐reported cognitive problems after delirium in the intensive care unit and the effect of systemic inflammation. *Journal of the American Geriatrics Society*.

[23] Bergeron N., Dubois M. J., Dumont M., Dial S., Skrobik Y. (2001). Intensive care delirium screening checklist: evaluation of a new screening tool. *Intensive Care Medicine*.

[24] George C., Nair J. S., Ebenezer J. A. (2011). Validation of the Intensive Care Delirium Screening Checklist in nonintubated intensive care unit patients in a resource-poor medical intensive care setting in South India. *Journal of Critical Care*.

[25] Detroyer E., Timmermans A., Segers D. (2020). Psychometric properties of the intensive care delirium screening checklist when used by bedside nurses in clinical practice: a prospective descriptive study. *BMC Nursing*.

[26] Knaus W. A., Draper E. A., Wagner D. P., Zimmerman J. E. (1985). Apache II: a severity of disease classification system. *Critical Care Medicine*.

[27] Munyua B. K. (2018). *Evaluating the Validity of Apache Ii as a Predictor of Icu Mortality for the Critically Ill Patients at Knh’scritical Care Units*.

[28] Vincent J.-L., Moreno R., Takala J. (1996). The SOFA (Sepsis-related Organ Failure Assessment) score to describe organ dysfunction/failure. *Intensive Care Medicine*.

[29] Laimoud M., Alanazi M. (2020). The Validity of SOFA Score to Predict Mortality in Adult Patients with Cardiogenic Shock on Venoarterial Extracorporeal Membrane Oxygenation. *Critical Care Research and Practice*.

[30] Le Gall J.-R., Lemeshow S., Saulnier F. (1993). A new simplified acute physiology score (SAPS II) based on a European/North American multicenter study. *JAMA*.

[31] Kądziołka I., Swistek R., Borowska K., Tyszecki P., Serednicki W. (2019). Validation of Apache II and SAPS II scales at the intensive care unit along with assessment of SOFA scale at the admission as an isolated risk of death predictor. *Anaesthesiology Intensive Therapy*.

[32] Sun D., Ding H., Zhao C. (2017). Value of SOFA, Apache IV and SAPS II scoring systems in predicting short-term mortality in patients with acute myocarditis. *Oncotarget*.

[33] Mossello E., Baroncini C., Pecorella L. (2020). Predictors and prognosis of delirium among older subjects in cardiac intensive care unit: focus on potentially preventable forms. *European Heart Journal: Acute Cardiovascular Care*.

[34] Xing J., Yuan Z., Jie Y., Liu Y., Wang M., Sun Y. (2019). Risk factors for delirium: are therapeutic interventions part of it?. *Neuropsychiatric Disease and Treatment*.

[35] Limpawattana P., Panitchote A., Tangvoraphonkchai K. (2016). Delirium in critical care: a study of incidence, prevalence, and associated factors in the tertiary care hospital of older Thai adults. *Aging & Mental Health*.

[36] Chen H., Jiang H., Chen B. (2020). The incidence and predictors of postoperative delirium after brain tumor resection in adults: a cross-sectional survey. *World Neurosurgery*.

[37] Norkienė I., Ringaitiene D., Kuzminskaite V., Sipylaite J. (2013). Incidence and risk factors of early delirium after cardiac surgery. *BioMed Research International*.

[38] Pan Y., Yan J., Jiang Z., Luo J., Zhang J., Yang K. (2019). Incidence, risk factors, and cumulative risk of delirium among ICU patients: a case-control study. *International Journal of Nursing Science*.

[39] Sharma A., Malhotra S., Grover S., Jindal S. K. (2012). Incidence, prevalence, risk factor and outcome of delirium in intensive care unit: a study from India. *General Hospital Psychiatry*.

[40] Lin Y. J., Lin L. Y., Peng Y. C. (2020). Association of Glucose Variability with Postoperative Delirium in Acute Aortic Dissection Patients: An Observational Study. *Journal of Cardiothorac Surgery*.

[41] Ashraf A., Hashmi M., Raza A., Salim B., Faisal Khan M. (2020). Incidence, risk factors and outcome of delirium in a surgical intensive care unit of a tertiary care hospital. *Qatar Medical Journal*.

[42] Maldonado J. R. (2018). Delirium pathophysiology: an updated hypothesis of the etiology of acute brain failure. *International Journal of Geriatric Psychiatry*.

[43] Watson P. L., Ceriana P., Fanfulla F. (2012). *Delirium: is sleep important?* Best practice & research. *Best Practice & Research Clinical Anaesthesiology*.

[44] Honarmand K., Rafay H., Le J. (2020). A systematic review of risk factors for sleep disruption in critically ill adults. *Critical Care Medicine*.

[45] Tsuruta R., Nakahara T., Miyauchi T. (2010). Prevalence and associated factors for delirium in critically ill patients at a Japanese intensive care unit. *General Hospital Psychiatry*.

[46] Lee J., Chung S., Joo Y., Kim H. (2016). The major risk factors for delirium in a clinical setting. *Neuropsychiatric Disease and Treatment*.

[47] Wu C., Zhu Y., Li G. (2020). Incidence and Risk Factors of Delirium in ICU Patients. *International Journal of Clinical and Experimental Medicine*.

[48] Öztürk Birge A., Bedük T. (2018). The relationship of delirium and risk factors for cardiology intensive care unit patients with the nursing workload. *Journal of Clinical Nursing*.

[49] Aldemir M., Ozen S., Kara I. H., Sir A., Bac B. (2001). Predisposing factors for delirium in the surgical intensive care unit. *Critical Care*.

[50] Zhang W. Y., Wu W. L., Gu J. J. (2015). Risk factors for postoperative delirium in patients after coronary artery bypass grafting: a prospective cohort study. *Journal of Critical Care*.

[51] Siew E. D., Fissell W. H., Tripp C. M. (2017). Acute kidney injury as a risk factor for delirium and coma during critical illness. *American Journal of Respiratory and Critical Care Medicine*.

[52] Liu Z., Pang X., Zhang X., Cao G., Fang C., Wu S. (2017). Incidence and risk factors of delirium in patients after Type-A aortic dissection surgery. *Journal of Cardiothoracic and Vascular Anesthesia*.

[53] Liu M., Liang Y., Chigurupati S. (2008). Acute kidney injury leads to inflammation and functional changes in the brain. *Journal of the American Society of Nephrology*.

[54] Lahariya S., Grover S., Bagga S., Sharma A. (2014). Delirium in patients admitted to a cardiac intensive care unit with cardiac emergencies in a developing country: incidence, prevalence, risk factor and outcome. *General Hospital Psychiatry*.

[55] Berger E., Wils E. J., Vos P. (2020). Prevalence and management of delirium in intensive care units in The Netherlands: an observational multicentre study. *Intensive and Critical Care Nursing*.

[56] Saravana-Bawan B., Warkentin L. M., Rucker D., Carr F., Churchill T. A., Khadaroo R. G. (2019). Incidence and predictors of postoperative delirium in the older acute care surgery population: a prospective study. *Canadian Journal of Surgery*.

[57] Wang J., Ji Y., Wang N. (2018). Risk factors for the incidence of delirium in cerebrovascular patients in a Neurosurgery Intensive Care Unit: a prospective study. *Journal of Clinical Nursing*.

[58] Saint S., Lipsky B. A., Goold S. D. (2002). Indwelling urinary catheters: a one-point restraint?. *Annals of Internal Medicine*.

